# Aldosterone Response in Severe Hypokalemia and Volume Depletion: A Case Report and Review of the Recent Research

**DOI:** 10.1155/2016/2036503

**Published:** 2016-07-20

**Authors:** Keiko Kai, Naoto Tominaga, Daisuke Uchida, Nanae Fukai, Yumie Matsuura, Susumu Uda, Akio Yokochi

**Affiliations:** Division of Nephrology, Kanto Rosai Hospital, Kanagawa 211-8510, Japan

## Abstract

We report a case of severe hypokalemia and volume depletion complicated by chronic watery diarrhea resulting from chronic alcoholism in a 57-year-old man. Prompt replacement of normal saline with potassium chloride and cessation of alcohol intake resulted in a favorable outcome. We discuss the pathophysiology of the case, emphasizing the response of aldosterone in both hypokalemia and volume depletion, and provide a review of recent research.

## 1. Introduction

Hypokalemia is an electrolyte abnormality commonly encountered in daily clinical practice. It can result from reduced potassium intake, potassium shift into cells, and/or extrarenal or renal potassium loss [1]. Diarrhea, vomiting, and excess sweating can cause extrarenal potassium loss [1], in which volume depletion can occur simultaneously. Because volume depletion sometimes affects vital signs (e.g., blood pressure), protective mechanisms are essential. Aldosterone, an adrenocortical hormone, plays a pivotal role in responding to volume depletion and hypotension, which maintains the homeostasis and hemodynamics of the body. In addition, aldosterone increases in hyperkalemia and promotes urinary potassium excretion. However, the mechanism of aldosterone response in conditions with both volume depletion and hypokalemia has not been clarified. Here, we report a case of severe hypokalemia and volume depletion complicated by chronic watery diarrhea resulting from chronic alcoholism.

## 2. Case Presentation

The patient was a 57-year-old man with a history of chronic myelogenous leukemia, hypertension, dyslipidemia, colon polyp (tubular adenoma, low-grade malignancy), and bilateral lower leg amputations due to a burn. He was also a heavy drinker and presented with chronic mild watery diarrhea. He had started experiencing bilateral upper extremity weakness and numbness, which gradually deteriorated. Two weeks later, he could not move by himself, and he was admitted to the hospital. His daily medications included imatinib 400 mg/day, valsartan 80 mg/day, atenolol 50 mg/day, eperisone 150 mg/day, ranitidine 300 mg/day, and irsogladine 1.5 g/day, but he had not taken them for several days before admission.

On admission, his blood pressure was 154/100 mmHg, pulse rate was 106 beats/minute in the supine position, and arterial oxygen saturation was 100% on room air. On physical examination, he showed dry mouth, hypoactive bowel sounds, weakening of tendon reflexes, 3/3 on a manual muscle test, a right-handed squeeze of 6 kg, a left-handed squeeze of 3 kg, and pain with pressure at the femurs; he did not show jugular vein distension, edema, or ascites. Electrocardiography revealed ST depletion, a tall U wave, and QTc prolongation. Laboratory data revealed serum concentrations of sodium of 140 mmol/L, chloride of 92 mmol/L, potassium of 2.0 mmol/L, corrected calcium of 2.2 mmol/L, phosphorus of 0.36 mmol/L, and magnesium of 1.2 mmol/L, with a serum anion gap of 10.2 mmol/L. Serum urea nitrogen and creatinine concentrations were 4.6 mmol/L and 61.0 *μ*mol/L, respectively. Other serum biochemistry values were as follows: aspartate aminotransferase level, 622 IU/L; alanine aminotransferase level, 116 IU/L; lactate dehydrogenase level, 994 IU/L; creatine kinase level, 20,340 IU/L (CK-MM, 94%); and urine occult blood reaction, strongly positive; urinary erythrocytes were not identified. Arterial blood gas analysis showed a pH level of 7.586, carbon dioxide tension of 44.1 mmHg, and bicarbonate level of 37.8 mmol/L, compatible with metabolic alkalosis. Urine electrolyte levels of sodium, potassium, and chloride were 50 mmol/L, 6.4 mmol/L, and 66 mmol/L, respectively, with a urine anion gap of −9.6 and pH level of 6.0. Levels of thyroid-stimulating hormone, free T4, adrenocorticotropic hormone, and serum cortisol were 1.51 mIU/L (normal, 0.35–4.94 mIU/L), 0.15 pmol/L (normal, 0.09–0.19 pmol/L), 4.33 pmol/L (normal, 1.58–13.93 pmol/L), and 14.6 *μ*g/dL (normal, 4.0–18.3 *μ*g/dL), respectively, in the early morning. Plasma renin activity and plasma aldosterone concentration (PAC) were 11 *μ*g/L/h (normal, 0.3–2.9 *μ*g/L/h) and 0.72 nmol/L (normal, 0.83–4.40 nmol/L), respectively, in the supine position. The transtubular potassium gradient was 2.9 in pOsm 288 mOsm/kgH_2_O and uOsm 319 mOsm/kgH_2_O, with fractional excretion of potassium of 2.1%, sodium of 0.24%, and urea nitrogen of 15.6%.

**Figure 1:**
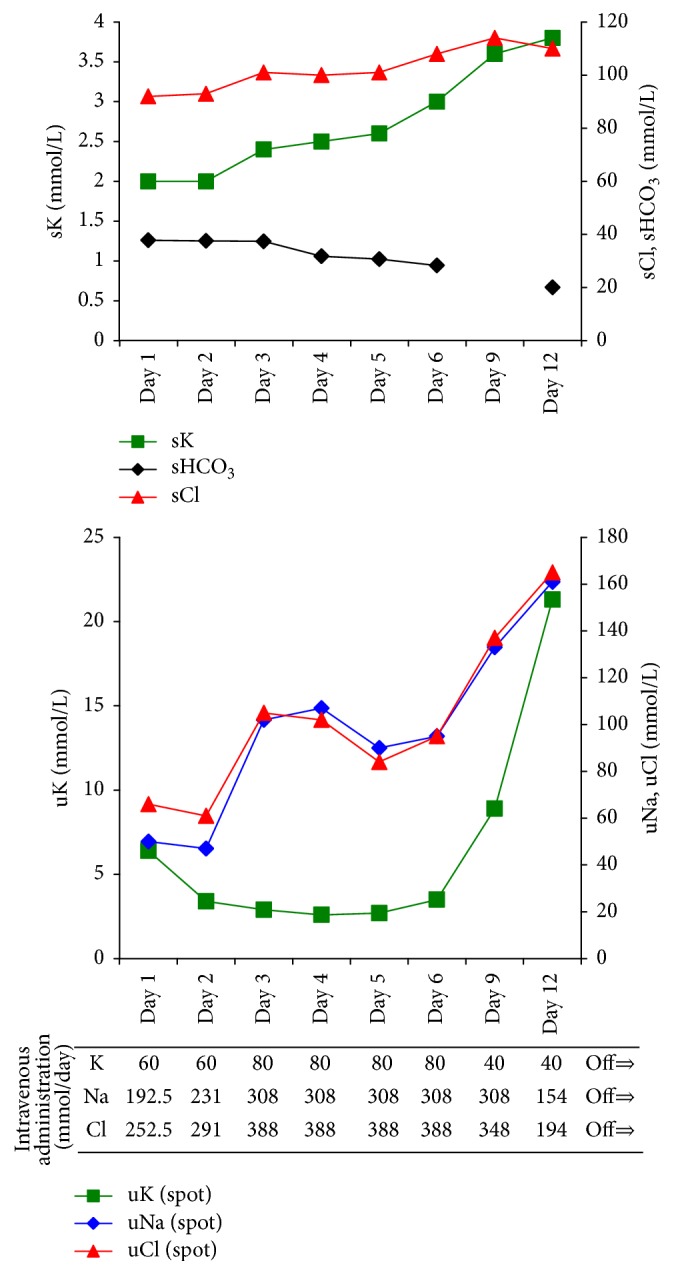
Clinical course. sCl, serum chloride; sHCO_3_, serum bicarbonate; sK, serum potassium; uCl, urinary chloride; uK, urinary potassium; uNa, urinary sodium.

A diagnosis of severe hypokalemia and volume depletion due to chronic watery diarrhea and concomitant chronic poor oral ingestion, complicated by rhabdomyolysis, was made. The patient received standard doses of potassium chloride (40–80 mmol/day) with normal saline intravenously in order to improve his serum potassium concentration and volume depletion. The clinical course is shown in Figure 1. His muscle weakness gradually improved over the clinical course. The mild watery diarrhea also gradually improved, and he was able to intake orally. After discharge on hospital day 23, the patient's serum potassium concentration was maintained within its normal range.

## 3. Discussion

Hypokalemia can result from inadequate potassium intake, shift of potassium from extracellular to intracellular fluid, and/or renal or gastrointestinal potassium loss [2]. In this patient, the main cause of hypokalemia seemed to be gastrointestinal loss because renal potassium conservation was observed and urinary potassium excretion appeared to be <20 mmol/day. Concomitant inadequate intake also could have contributed to hypokalemia; inadequate intake by itself is rarely a cause if the kidneys are able to reduce potassium excretion to approximately 10 mmol/day. There are 3 important factors that affect renal potassium excretion: (1) increased mineralocorticoid receptor (MR) stimulation, (2) increased distal sodium delivery, and (3) increased nonreabsorbable ions in distal nephrons [1].

Metabolic alkalosis also was seen in this patient, which is usually associated with potassium loss from the upper gastrointestinal tract, such as with vomiting or nasogastric drainage, and is not usually seen with diarrhea or laxative abuse. The pathophysiology of metabolic alkalosis consists mainly of 2 parts: (1) loss of hydrogen and (2) increase of bicarbonate. The primary causes of the maintenance of metabolic alkalosis include (1) reduction of effective circulating volume, (2) chloride deficiency, (3) hypokalemia, and (4) decreased renal function [3–5]. In the present case, the main cause of metabolic alkalosis may have been volume depletion or chloride deficiency. In hypokalemia, reabsorption of bicarbonate increases in the proximal tubules, while, in chloride deficiency, secretion of bicarbonate decreases in *β*-intercalated cells of the cortical collecting tubules.

In the present case, the kidneys seemed to be responding to hypokalemia normally based on the low PAC. However, because the patient simultaneously showed volume depletion, the renin-angiotensin-aldosterone system (RAAS) should have been upregulated in order to maintain body fluid status. Generally, when aldosterone increases, serum potassium decreases, because it promotes secretion in the distal tubules. In 1977, through a study of hemodialysis patients, Henrich et al. showed that the effect of potassium depletion on inhibiting aldosterone secretion was more potent than the effect of volume depletion and/or hypotension on promoting aldosterone secretion [6]. There were two different dialysis protocols. In one, 5 patients underwent 7 dialyses, and the dialysate potassium concentration was adjusted to maintain the plasma potassium level so that it did not change by >0.3 mmol/L. In another group of 12 dialyses involving 8 patients (including the original 5 patients), decreases in the plasma potassium level were permitted. In both groups, the goal was weight loss by ultrafiltration and all patients lost at least 0.5 kg. However, despite a comparable amount of volume depletion and a significant increase in plasma renin activity, a decrease in PAC occurred with the hypokalemic dialyses, from 3.32 ± 1.11 nmol/L to 1.02 ± 0.50 nmol/L (*P* < 0.005) [6]. However, this study did not address the mechanism of aldosterone response. In addition, the mechanism of aldosterone response in conditions with both volume depletion and hypokalemia remains unclear.

**Figure 2:**
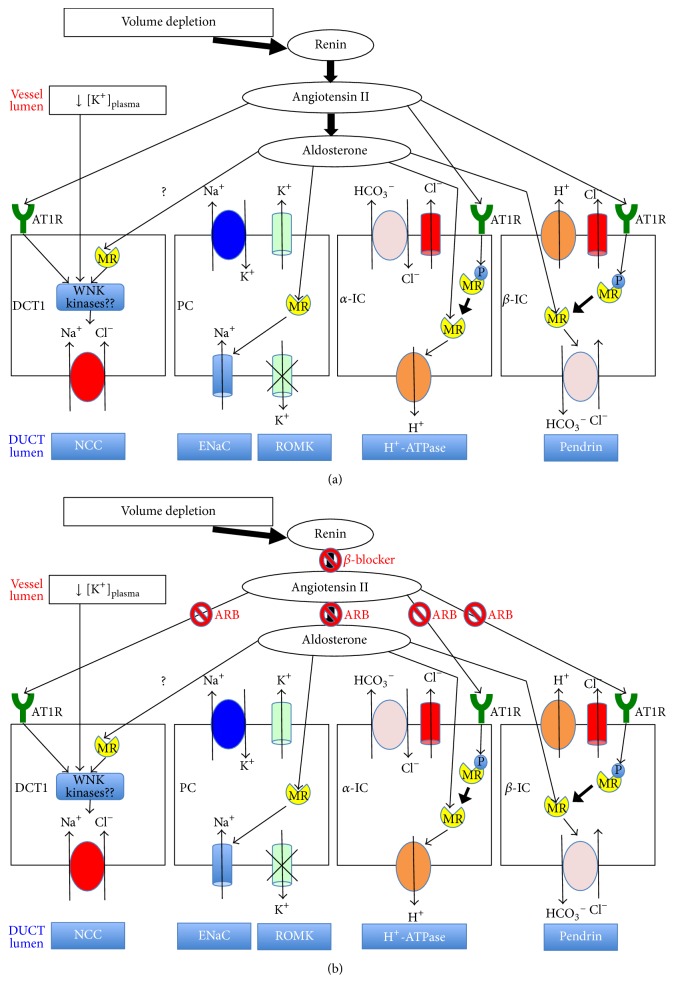
(a) Angiotensin II and aldosterone response in volume depletion and hypokalemia. (b) Angiotensin II and aldosterone response are affected by both an angiotensin II type 1 receptor blocker and a *β*-adrenoreceptor antagonist. *α*-IC, *α*-intercalated cell; ARB, angiotensin II type 1 receptor blocker; AT1R, angiotensin II type 1 receptor; *β*-blocker, *β*-adrenoreceptor antagonist; *β*-IC, *β*-intercalated cell; DCT1, initial part of the distal convoluted tubules; ENaC, amiloride-sensitive sodium channel; MR, mineralocorticoid receptor; NCC, thiazide-sensitive NaCl cotransporter; P, phosphorylated; PC, principal cell; ROMK, renal outer medullary potassium channel; WNK, with-no-lysine [K].

The ability of aldosterone to signal the kidneys to stimulate sodium retention without potassium secretion in volume depletion and to stimulate potassium secretion without sodium retention in hyperkalemia has been referred to as the aldosterone paradox [7]. In 2013, Shibata et al. showed the precise mechanism of the aldosterone paradox [8]. There are mainly 2 types of cells in the renal collecting ducts: principal cells and intercalated cells. The epithelial sodium channel and renal outer medullary potassium channel are expressed in the apical membrane of principal cells. H^+^-ATPase is expressed in *α*-intercalated cells, while pendrin, a chloride-bicarbonate exchanger, is expressed in *β*-intercalated cells. Interestingly, MRs are expressed in all cells, but serine 843 is phosphorylated only in MRs in intercalated cells. In volume depletion, MRs in intercalated cells are dephosphorylated by angiotensin II (ANG2). Thus, in addition to MR signaling in principal cells, ANG2 signaling reduces phosphorylated serine 843, resulting in MR signaling activation in intercalated cells. This promotes chloride reabsorption via both the apical H^+^-ATPase and the apical chloride-bicarbonate exchanger pendrin. The increase in chloride reabsorption results in a lumen with neutral potential, which prevents increased potassium efflux [8] (Figure 2(a)). Moreover, several clinically relevant hormones stimulate sodium reabsorption in the initial part of the distal convoluted tubules (DCT1) by increasing thiazide-sensitive NaCl cotransporter (NCC) activity. One of these hormones is ANG2 [9, 10] (Figure 2(a)). Some investigators reported that aldosterone may also regulate NCC via MR [11, 12], whereas others reported that it may not regulate NCC via MR directly [13–15]. Though the action of aldosterone remains controversial (Figure 2(a)), it would be possible for PAC to increase and for urinary sodium and chloride to decrease.

Interestingly, our patient had been taking an ANG2 type 1 receptor blocker (ARB) and a *β*-adrenoreceptor antagonist (*β*-blocker) for hypertension until a few days before admission. This might have made the pathophysiology more complicated because they may have affected the response of the RAAS. By competitively blocking ANG2 to act on its receptors, MRs could not be fully dephosphorylated by ANG2 in intercalated cells. In addition, ARBs and *β*-blockers inhibit aldosterone production, which affects aldosterone action in DCT1, principal, and intercalated cells. As a result, urinary sodium and chloride exhibited much higher levels than expected in this patient (Figure 2(b)). Meanwhile, urinary potassium was normally suppressed, probably due to both low PAC and volume depletion. In 2015, Terker et al. [16] showed that plasma potassium concentration signals to the NCC by altering intracellular chloride concentration in the DCT. DCT cells are exceptionally sensitive to changes in plasma potassium concentration. Thus, when plasma potassium concentration is low, intracellular chloride concentration is low, the with-no-lysine [K] kinases may be turned on, and NCC is activated. Moreover, low plasma potassium concentration itself does not influence aldosterone secretion (Figures 2(a) and 2(b)).

In summary, it is difficult to comprehend the mechanism of aldosterone response in conditions with both volume depletion and hypokalemia. Recent research has been helpful, but management of patients is still difficult considering the factors that may affect the RAAS in hypokalemia and volume depletion.
